# Multi-Omics Integrative Approach of Extracellular Vesicles: A Future Challenging Milestone

**DOI:** 10.3390/proteomes10020012

**Published:** 2022-04-22

**Authors:** Enxhi Shaba, Lorenza Vantaggiato, Laura Governini, Alesandro Haxhiu, Guido Sebastiani, Daniela Fignani, Giuseppina Emanuela Grieco, Laura Bergantini, Luca Bini, Claudia Landi

**Affiliations:** 1 Functional Proteomics Lab, Department of Life Sciences, University of Siena, 53100 Siena, Italy; lorenz.vantaggiato@student.unisi.it (L.V.); luca.bini@unisi.it (L.B.); landi35@unisi.it (C.L.); 2 Department of Molecular and Developmental Medicine, University of Siena, 53100 Siena, Italy; laura.governini@unisi.it (L.G.); alesandro.haxhiu@student.unisi.it (A.H.); 3 Diabetes Unit, Department of Medicine, Surgery and Neurosciences, University of Siena, 53100 Siena, Italy; guido.sebastiani@unisi.it (G.S.); dfignani@gmail.com (D.F.); giusy.grieco.90@gmail.com (G.E.G.); 4 Fondazione Umberto Di Mario, c/o Toscana Life Sciences, 53100 Siena, Italy; 5 Respiratory Diseases and Lung Transplant Unit, Department of Medical Sciences, Surgery and Neurosciences, University of Siena, 53100 Siena, Italy; laurabergantini@gmail.com

**Keywords:** multi-omics, proteomics, transcriptomics, metabolomics, lipidomics, surfaceomics, system biology, EVs origin

## Abstract

In the era of multi-omic sciences, dogma on singular cause-effect in physio-pathological processes is overcome and system biology approaches have been providing new perspectives to see through. In this context, extracellular vesicles (EVs) are offering a new level of complexity, given their role in cellular communication and their activity as mediators of specific signals to target cells or tissues. Indeed, their heterogeneity in terms of content, function, origin and potentiality contribute to the cross-interaction of almost every molecular process occurring in a complex system. Such features make EVs proper biological systems being, therefore, optimal targets of omic sciences. Currently, most studies focus on dissecting EVs content in order to either characterize it or to explore its role in various pathogenic processes at transcriptomic, proteomic, metabolomic, lipidomic and genomic levels. Despite valuable results being provided by individual omic studies, the categorization of EVs biological data might represent a limit to be overcome. For this reason, a multi-omic integrative approach might contribute to explore EVs function, their tissue-specific origin and their potentiality. This review summarizes the state-of-the-art of EVs omic studies, addressing recent research on the integration of EVs multi-level biological data and challenging developments in EVs origin.

## 1. Introduction

In the era of multi-omic sciences, which continuously provide a more comprehensive overview of human pathogenesis, dogma on singular cause-effect in physio-pathological processes is overcome and system biology approaches have been welcomed to look at pathologies through new perspectives [1,2,3,4,5]. In this context, extracellular vesicles (EVs) have also been targets of this approach, shedding light on their heterogeneity in terms of content, function, origin and potentiality [6]. EVs are phospholipid bilayer-enclosed vesicles released by all cell types, and they can be found in several biological fluids such as saliva [7], breast milk [8], tears [9], bronchoalveolar lavage fluid [10], blood [11], urine [12], cerebrospinal fluid [13,14] and others. They can be classified according to their size, biogenesis and function, however they are mainly distinguished into three classes: exosomes, which form by inward invagination of the endosomal membrane, then released into the extracellular space by fusion of the so-called “multivesicular body (MVB)” with the plasma membrane and range from 20 to 150 nm in size; microvesicles or ectosomes, which originate from outward budding of the plasma membrane and range from 100 to 1000 nm in size; then, apoptotic bodies, which form during apoptosis by outward budding of the plasma membrane, ranging in size from 100–5000 nm [15,16]. As EVs knowledge is continuously growing and evolving, their classification has become more difficult to be established, therefore, their size and biogenesis are no longer sufficiently useful criteria to differentiate them [6]. Thus, alternative criteria were explored for EVs classification, relying rather on their function. Their principal functional role is mediators of intercellular communication as they carry cargos from their cell of origin and deliver to specific recipient cells; indeed, their content is widely heterogeneous as they pack DNA, RNAs, proteins, lipids and metabolites [17,18]. For this reason, the widespread idea that EVs are just mediators of communication has no longer been accepted as they are instead functionally active acting as signal transducers. For example, they mediate antigen presentation modulating immune responses [19] and participate in several biological processes, such as tissue regeneration [20], autoimmunity [21,22], vascularization [23] and metabolic reprogramming [24] by transferring their content. As a result, EVs might be considered as proper biological systems whose content and function not only echo their cell of origin, but also generate from the direct response of the cell to various stimuli, thereby they act as mediators of those responsive signals both at local and systemic level [6,13,25]. Given this complex EVs heterogeneity, omic sciences are optimal tools that allow to unravel EVs multi-level biological data with the purpose of deeper understanding of their functions and, above all, of their potentialities [6]. Looking deeper in their molecular content, both circulating and tissue-specific EVs are being used as diagnosis and prognosis biomarkers at every level of content. Several diagnostic markers are being identified at RNA, protein and metabolic level of EVs in a wide range of cancers [26], such as colorectal cancer [27,28], prostatic cancer [29,30], lung cancers [31,32], breast cancer [33,34], as well as in many other diseases, such as pulmonary diseases [35,36,37], neurodegenerative diseases [38,39] and others. In fact, the most pursued objective in EVs biomarker discovery is the diagnosis of early-stage diseases by circulating vesicles from blood, urine or plasma, referred to as “liquid-biopsy”, as easily accessible biological fluids [40]. Furthermore, EVs also possess a powerful therapeutic potential as they could be used as direct therapeutics or loaded with active pharmaceutical molecules [41,42,43,44,45]. These potentialities culminate in the development of promising novel technologies such as outer membrane vesicles (OMVs)-based vaccines, consisting of naturally released vesicles from Gram-negative bacteria containing key antigenic components required to elicit a protective immune response [46]. This led the way to studies on novel biotechnological innovations, such as nanovaccines against various types of cancers [47] and nano-based therapies [48,49].

## 2. Extracellular Vesicle as Resourceful Biological Systems

As intricated heterogeneous structures, EVs’ first approach is their content profiling, characterizing as much as possible their biomolecules, such as proteins, RNAs, metabolites and lipids. Indeed, their proper characterization is the fundamental requisite for understanding the EVs’ functions. However, EVs content characterization is beyond the focus of this review, rather it is to consider omic sciences potentialities in EVs functions and applications.

### 2.1. Proteomics

**Figure 1:**
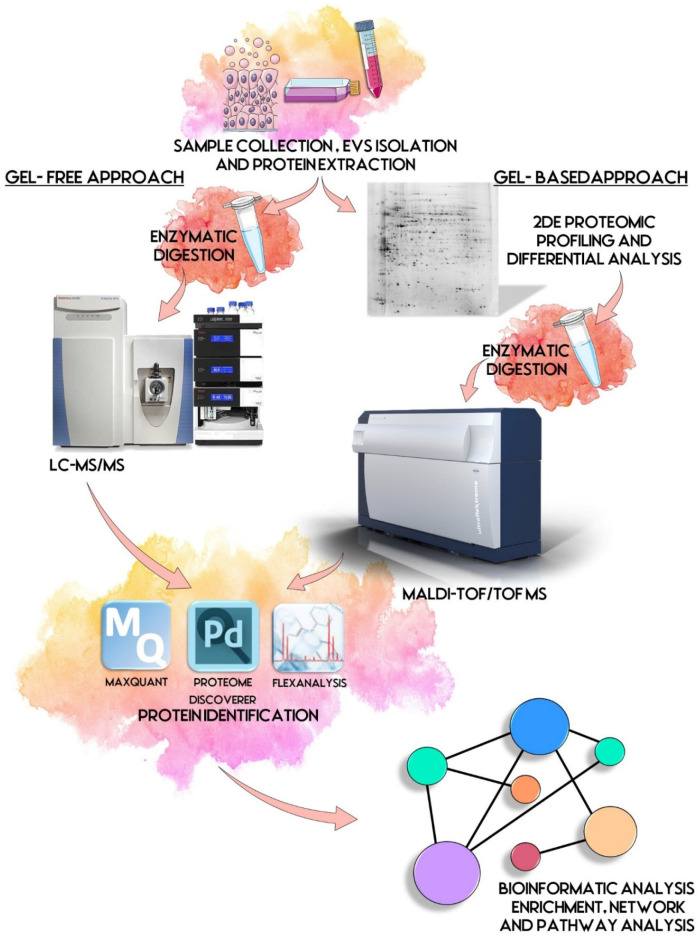
EVs proteomic workflow. First step is the sample collection and EVs isolation starting commonly from biological fluids and cell lines. Proteins are extracted and prepared for proteomic analysis. By gel-based approach, a 2DE proteomic profiling and differential analysis is performed, followed by acquisition of mass spectra by MALDI-TOF/TOF, and protein identification. By gel-free approach, proteins are digested enzymatically and processed by LC-MS/MS and dedicated software. Identified proteins are then subjected to biological interpretation by bioinformatic analysis, such as enrichment, network and pathway analyses. Some illustrations were adapted from Servier Medical Art, licensed under a Creative Commons Attribution 3.0 Unported License.

EVs proteomics explicates its potentiality as it might suggest numerous answers to tricky questions, following well-defined protocols and taking advantage of many different approaches, such as gel-free and gel-based (Figure 1).

**Figure 2:**
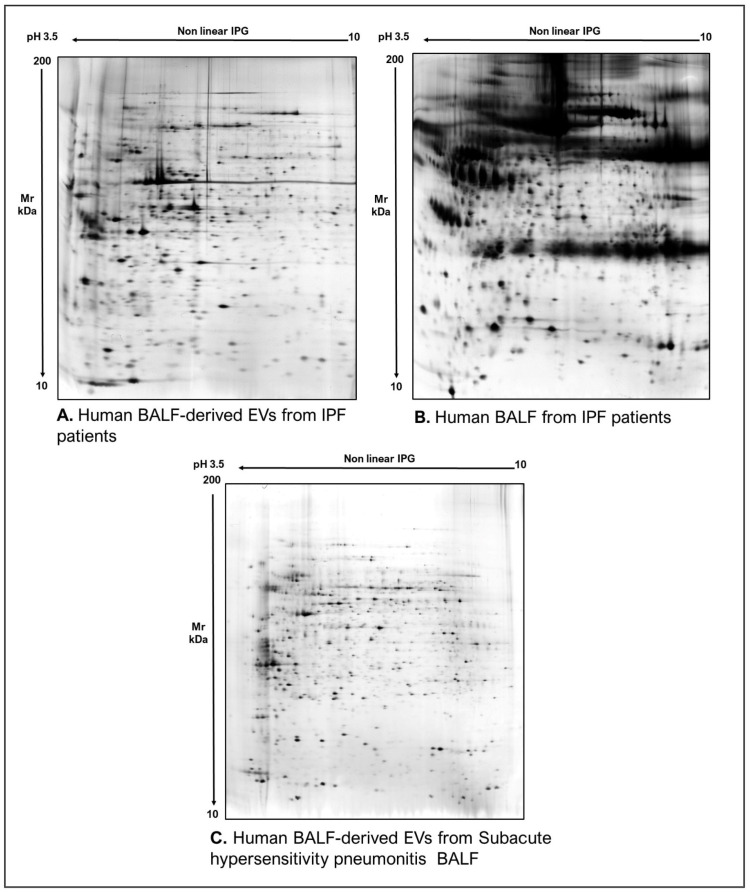
Two-dimensional electrophoresis images indicative of the protein profile of (**A**) EVs from BAL of IPF patients, (**B**) BAL of IPF patients and (**C**) EVs from BAL of subacute hypersensitivity pneumonitis patients.

**Figure 3:**
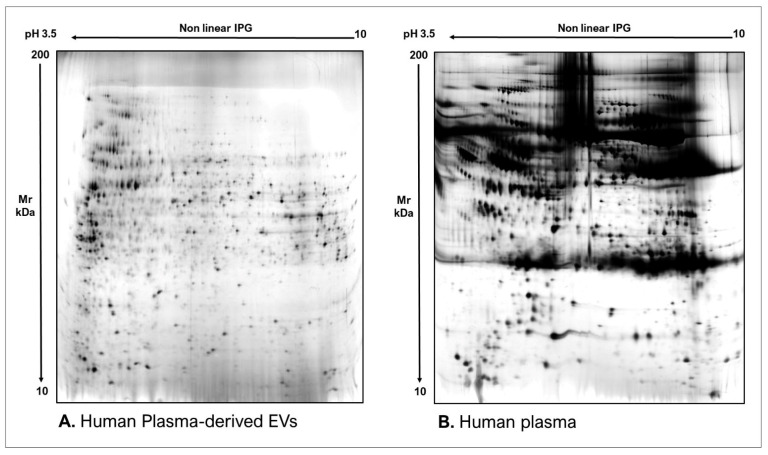
Two-dimensional electrophoresis images indicative of the protein profile of (**A**) Astrocytes culture-derived EVs and (**B**) astrocytes cells.

**Figure 4:**
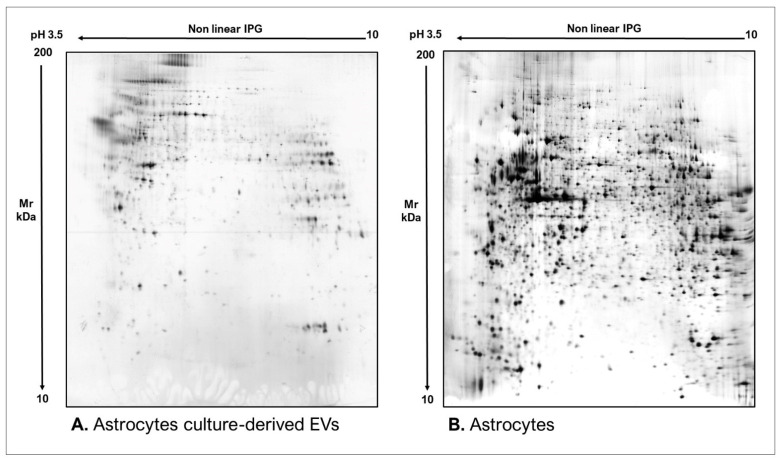
Two-dimensional electrophoresis images indicative of the protein profile of (**A**) human plasma-derived EVs and (**B**) human plasma.

**Figure 5:**
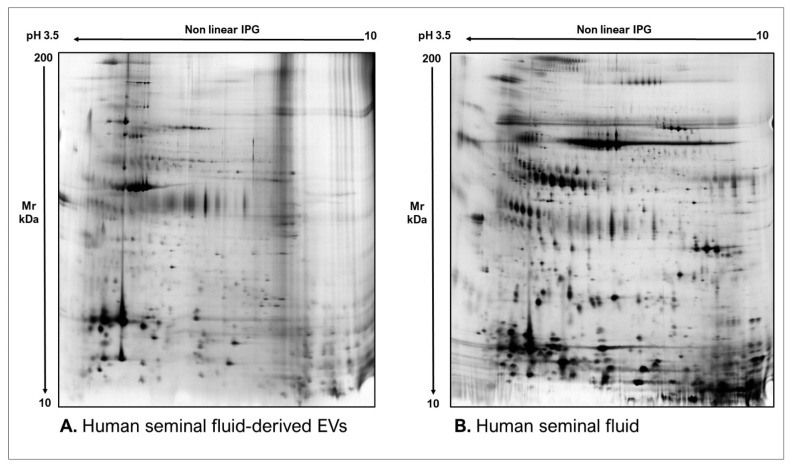
Two-dimensional electrophoresis images indicative of the protein profile of (**A**) Human seminal fluid-derived EVs and (**B**) Human seminal fluid.

As commonly accepted, EVs’ heterogeneity in term of subtypes is far beyond the classical classification by size or biogenesis and the difficulty to discriminate them within a whole biological sample represents a limit in analyzing their protein cargoes, how these change across the subpopulations and how these associate with a pathogenic process [50]. In the recent years, there have been various attempts to comparatively analyze the protein cargo of different EVs populations, often comparing small-EVs (s-EVs) and large-EVs (l-EVs). Although several common proteins are identified, numerous unique proteins are also detected, primarily suggesting potential subtypes markers and distinct and/or common associated molecular pathways [51,52]. Indeed, getting deeper into EVs populations is not just limited to their classification purpose, but it also extends to investigate their biological variability. Haraszti et al. reported a comparative proteomic study of s-EVs and l-EVs isolated from glioblastoma, hepatocellular carcinoma and bone marrow mesenchymal stem cells (MSC). The proteomic data of s-EVs resulted closely related to the donor cells proteome, rather than the l-EVs one, allowing to differentiate cancer cells and MSC [53,54]. As the proteomic profile of EVs is not only related to the vesicular main components, rather it includes an extensive part of proteins strictly related to their progenitor cells, the proteomic approach by two-dimensional electrophoresis (2DE) might retain a valuable potentiality. In our recent paper about the proteomic characterization of EVs from bronchoalveolar lavage fluid (BALF) of idiopathic pulmonary fibrosis (IPF) patients, we compared the 2DE map of EVs isolated from BALF with the 2DE image of the whole biological sample, showing that the obtained protein profiles were different and characteristics [55]. As Figure 2 shows, the 2DE proteomic pattern of IPF BALF-EVs (Figure 2A) is visually unique and dissimilar to that of the whole BALF (Figure 2B), as well as to that of EVs isolated from the BALF of patients with other interstitial lung diseases (ILDs) (Figure 2C). These results first confirm the good isolation of EVs as any or very few contaminants, such as highly abundant protein species normally present in common biological fluids, are visible in the vesicular 2DE maps; secondly, they underline the strong divergent protein content between the whole biological sample and the derived EVs. Remarkably, other proteomic analyses conducted by our research group highlighted how the vesicular protein cargo is distinct from its original sample; Montecchi et al. focused on the differential proteomic analysis by 2DE of murine astrocytes and murine astrocytes-derived EVs [56]. As Figure 3 shows, the 2DE vesicular map (Figure 3A) is clearly different from the proteomic pattern of the cellular culture of origin (Figure 3B) and highly characteristic of its original sample. Furthermore, our research group performed other studies on EVs isolated by different biological samples. In Figure 4A,B, it can be observed the 2DE map of plasma-derived EVs, isolated by a combination of size exclusion chromatography (SEC) and a commercial isolation kit, and of human plasma, respectively. Moreover, Figure 5A,B shows the 2DE proteomic profile of human seminal plasma-derived EVs by commercial isolation kit and of human seminal plasma, respectively. Even through different isolation procedures, the unique proteomic profile of EVs might be observed as clearly distinct from that of the original sample in all of our studies, as well as distinct also between themselves, suggesting 2DE as a good visualization technique of vesicular proteomes and of their correlation to their belonging progenitor sample. Other studies used proteomic approach by 2DE to perform EVs profiling, such as Di Giuseppe et al. in the characterization of two different EVs subpopulations isolated from human glioblastoma stem cell secretome [57], as well as Kitamura et al., who performed a proteomic profiling of exosomes from blood samples of patients affected by Parkinson’s disease [58].

Of equal importance, mass spectrometry by shotgun approach might provide a wide and highly sensitive range of information on EVs proteomic content, contributing considerably to their characterization and profiling [59,60,61,62,63]. Furthermore, this high-throughput approach has been continuously demonstrating its potentiality in EVs biomarkers discovery, as well as their therapeutical applications [64,65]. For instance, another important application of EVs proteomic profiling is the identification of disease-associated diagnostic and prognostic markers, as EVs proteome is both cell and disease-type dependent. Indeed, EVs are also capable of shaping the microenvironment as active players also in pathological events, such as metastatic promotion and tumor growth promotion [66,67]. The active involvement of EVs in cancer homeostasis and the fact that their composition reflects the contents of cell of origin make them optimal candidates for biomarker profiling studies. Liquid biopsies, indeed, are gaining great popularity given their many advantages in disease diagnosis, and proteomics retains a fundamental role as EVs protein biomarkers allow to overcome limits given by abundant proteins that dominate common biological fluids. For example, Choi et al. reported the identification of CD5L in serum-derived EVs as lung cancer biomarker [68], while Ganig et al. reported QSOX1 as a promising novel EVs biomarker for early diagnosis of colorectal cancer [69]. Other studies focused also on EVs biomarker profiling in other pathologies, such as Nielsen et al. who explored serum-EVs protein biomarkers for Alzheimer’s disease diagnosis [70]. Furthermore, EVs proteome could offer a snapshot of proteomic signatures reflecting on one side diverse stages of diseases progression; for example, recently Pecankova et al. reported a specific clusterin proteoform as a promising biomarker for myelodysplastic syndrome [71], and on the other side mirroring a response to therapy [72,73]. EVs proteomics also contributes considerably to EVs application as drug delivery systems. In fact, therapeutical agents or biological cargo may be encapsulated into EVs and then the vesicles engineered to acquire the requested features for delivery. Moreover, EVs are preferred rather than nanoparticles or liposomes because of their nano size, their ability to cross biological barriers and reduced immunogenicity [74]. Haney et al. report many studies on the use of EVs-carriers for particular therapeutic agents, such as paclitaxel and doxorubicin for the treatment of pulmonary metastatic cancer and triple-negative breast cancer, and also catalase and tripeptidyl-peptidase-1 (TPP1) for the treatment of Parkinson’s disease [75]. Remarkably, a more comprehensive understanding of EV proteomes and how their protein composition is influenced physio-pathologically by various factors could enable a more efficient exploitation of engineering strategies for the delivery of therapeutics in future [76]. Summary data are reported in Table A1.

### 2.2. Transcriptomics

EVs RNA content has been considerably investigated in the past years, reporting the presence of prevalently mRNAs, miRNAs and long non-coding (lnc) RNAs. However, the development of more advanced high-throughput RNA sequencing methods has led to the detection of various other RNA species, such as small nuclear (sn) RNA, small nucleolar (sno) RNA, non-coding (nc) RNA, long intergenic non-coding (linc) RNA, piwi-interacting (pi) RNA, vault RNA, small non-coding (Y)-RNA, small conditional (sc) RNA, circular (circ) RNA, signal recognition particle (SRP)-RNA and 7SK-RNA, as well as fragments originating from rRNA, tRNA, mRNA and lncRNAs [77,78,79]. Given the variety of functions and activities exploited by EVs RNAs, they offer a great chance to determine novel potential biomarkers of screening, diagnosis and prognosis of pathological conditions. For instance, Rhode et al. investigated the expression level of mRNAs encoding for CD44, PTEN and FASN in plasma-derived EVs from patients with gastric cancer, in order to improve differential diagnosis of different subtypes of this tumor [80]. Newman et al. investigated the potentiality of four specific miRNAs in the differential diagnosis of non-alcoholic fatty liver disease (NAFLD) and non-alcoholic steatohepatitis (NASH) in plasma-derived EVs [81]. Nonetheless, several studies have been demonstrating that miRNAs represent just a minority of the total RNAs contained in EVs, throwing light on the classes of non-coding RNAs [82]. Recently, Iparraguirre et al. investigated the RNA cargo in plasma-derived EVs of multiple sclerosis (MS) patients, reporting a particular enrichment of circRNAs in EVs of MS patients and significant differences in circRNA and linear RNA expression between MS types as well [83]; Magaña et al. evaluated the small ncRNA pattern in EVs released from medulloblastoma (MB) and diffuse infiltrative pontine glioma (DIPG) patient-derived cell lines, identifying novel miRNAs not previously associated to the pathogenesis of the diseases and demonstrating an enrichment of Y-RNAs in the released EVs [84], and Peng et al. evaluated piRNAs in urinary EVs as non-invasive biomarkers for prostate cancer diagnosis [85]. Although scientific community has been giving great attention to the EVs RNA profiling in order to explore their gene-regulatory activities and their potentiality in paving a new direction in therapeutic targeting, the state-of-the-art of EVs transcriptomics is to provide a clear picture of the EVs content, however many improvements are needed to better understand the message mediated by these structures [86]. Summary data are reported in Table A1.

### 2.3. Metabolomics and Lipidomics

Despite growing potentiality of metabolomics, EV databases, such as Vesiclepedia, ExoCarta or EVpedia mainly contain protein, mRNA and miRNA entries with less lipid and metabolite data [87,88,89]. Actually, in the last decade metabolomics has been demonstrating its promising contribution to EVs research. Metabolites are represented by any biologically relevant molecule below 2 kDa in size, therefore a wide range of molecules are included, such as steroid hormones, metabolic intermediates, amino acids, nucleotides and different enzymatic cofactors. Its first application is the EVs profiling of the metabolomic content. EVs metabolome, indeed, represents a phenotype screenshot of progenitor’s cellular state, thereby it may provide helpful information on metabolic dysregulations occurring in physio-pathological events [90]. Furthermore, monitoring of changes in EVs metabolome in patient’s biofluids could be extremely useful in providing information on disease progression and response to treatments [91]. Of interest, the major contribution of EVs metabolomics is given by providing encouraging metabolite biomarker candidates. For example, Clos-Garcia et al. reported an elevated level of the steroid hormone dehydroepiandrosterone sulphate (DHEAS) in urinary EVs from prostate cancer patients [92]. In addition, recently Lou et al. identified four metabolites as potential markers for severe acute pancreatitis (SAP) diagnosis, including eicosatrienoic acid (C20:3), thiamine triphosphate, 2-Acetylfuran, and cis-Citral [93]. Beyond clinical applications, EVs metabolome might also provide alternative information on EVs as metabolically active machines. For example, some studies demonstrated the involvement of EVs in the metabolic regulation of the extracellular space, such as in tumor growth and metastatic processes [94]. Remarkably, there is a strong crossover between EV metabolomics and lipidomics, as the size of most relevant lipids identifies them as metabolites [95]. In particular, lipidomics focuses on the characterization and quantification of lipid species as functionally active molecules, as well as on the lipid compositional response to various stimuli. For such reasons, this omic science addresses first a more comprehensive understanding of EVs biogenesis and packaging pathways [96,97]. In particular, numerous EVs lipidomic studies demonstrated that EVs are rich in certain lipids, such as cholesterol, sphingomyelin, phosphatidylserine (PS), phosphatidylcholine (PC), and phosphatidylinositol (PI), suggesting their role as cell-to-cell lipid mediators [98,99]. On the other hand, EVs lipidomes’ characterization is valuable in evaluating lipid dysregulations associated to various diseases. For instance, Su et al. investigated the lipidomic profile of brain-derived EVs (BDEVs) in human frontal cortex to determine a potential altered lipid pattern in Alzheimer’s disease [100]. The other encouraging application of lipidomics is the research of EVs lipid biomarkers. For example, Zhao et al. investigated plasma derived-EVs lipidomes of women in the early second trimester with preterm and fully-term births [101], while Luo et al. focused on the identification of potential metabolites biomarkers in pleural effusion-derived EVs for the rapid and correct diagnosis of tuberculosis pleural effusion (TPE) and malignancy pleural effusion (MPE) through both a metabolomic and lipidomic approach [93]. Nowadays, EVs represent wonderful candidates for novel ground-breaking drug delivery systems and for this reason, EVs lipidomics deals with an important challenge. In fact, the lipid composition of EVs and the stability of their membrane structure make them very stable; however, issues on how to load therapeutic agents into these vesicles still remain. Given this, lipidomics might represent a great source of knowledge of EVs lipid composition and might provide valuable insights for EVs biotechnologies [99]. Summary data are reported in Table A1.

### 2.4. Genomics

Another branch of EVs omic research addresses their genomic cargo, including single strand (ss) DNA, double strand (ds) DNA, mitochondrial (mt) DNA, plasmid DNA, along with nuclear protein histones as well. In addition, DNA could be found either enclosed within EVs or attached to the EVs’ outer surface, or both [18]. The most promising application of EVs genomics is its biomarker use, as EVs DNA reflects the mutational status of parental cells, making the diagnosis and prognosis of different pathological conditions, such as tumors, easier and more accessible. This is supported also by the enhanced availability of EVs DNA in body fluids as protected from degradation by the EVs lipid bilayer [102]. Many examples are given by the extensive research on liquid biopsies of various cancers [103]; for instance, Takur et al. reported the detection of specific colon carcinoma-associated mutations in plasma-derived EV-DNA; Lee et al. tested the sensitivity in detecting epidermal growth factor receptor (EGFR) mutations in EVs-DNA from BALF of patients with advanced non-small cell lung cancer (NSCLC) by targeted next-generation sequencing (NGS) of both tissue DNA and vesicular DNA [104]. Moreover, various studies have evaluated the methylation profile of EVs DNA, proving similarities with the methylation pattern in tissue-derived DNA [102]. For instance, Maire et al. reported that EVs-DNA derived from glioblastoma cells reflects the tumor methylation pattern and mutational profiles of tumor tissue-derived DNA [105]. Of particular interest, EVs genomics shed light also on EVs engineering for therapeutical use, as several studies have been attempting at loading DNA into EVs, still encountering many technical limits [106,107]. Although the biggest goal is to explore the potentiality of these nanostructures in delivering chosen molecules to specific targets, still many questions remain to be elucidated on the natural EVs behaviour itself [108]. Summary data are reported in Table A1.

### 2.5. Surfaceomics

Among omic sciences, surfaceomics has been attracting much attention recently. Surfaceome consists of the collection of membrane proteins of which parts are exposed to the extracellular space, for this reason it might be considered responsible for the interaction between the cells and the surrounding environment. Its growing interest is associated to the several functions that the surfaceome carries out and that might be target of biotechnological innovations: first, it regulates the selective permeability of the plasma membrane, which is of particular importance for efficient drug delivery systems to cells; second, the surfaceome is rich in receptors that capture and mediate specific external signals to the internal compartment of the cell, triggering a specific response. Moreover, the surfaceome is also composed of proteins that mediates both a defense and offense against harmful attacks. Due to these features, the surfaceome is a big source of knowledge of physio-pathological processes, as well as of potential pharmaceutical targets [109]. For instance, Rose et al. reported the identification of potential therapeutical targets for the glioblastoma treatment, as they investigated the glioblastoma-associated surfaceome by shotgun proteomics [110]. Another recent innovative surfaceomics application focused on the identification of novel surface antigens in bacterial surfaceome for vaccine development, as reported by Luu et al. [111]. As a result, surfaceomics has found its application also on EVs research. On one side, EVs surfaceomics might provide candidate surface proteins for the enrichment of EVs derived from specific cell types, such as cancer-cell exosomes, enhancing the ability to differentiate EVs produced from diverse tissues and improving EVs-based diagnosis. For example, Castillo et al. carried out the surfaceome profiling of pancreatic ductal adenocarcinoma (PDAC) exosomes by proteomic approach [112]. On the other side, the study of EVs surfaceome might provide powerful tools for diagnostic and therapeutic actions, such as antibody mediated therapy, as well as for drug delivery strategies by engineering EVs with specific target-cell recognition protein on their surface [113,114]. Summary data are reported in Table A1.

### 2.6. EVs’ Origin

As previously discussed, omic biological data represent the most promising source of information of EVs. However, one of the most important points of EVs is still challenging to be determined: their origin. Being able to identify the specific tissue and/or cell type from which EVs in a certain biological fluid originate has many advantages: first, EVs from different sources own a unique signature content, at each omic level, and specific surface proteins which not only may allow to date back to their progenitor tissue or cell type, but also to determine specific biomarkers of local and systemic pathologies just by easily accessible biological samples, such as blood and plasma. Secondly, it may provide additional information on tissue crosstalk via EVs, contributing to the understanding of EVs functions at a paracrine and endocrine level [115]. Furthermore, it could contribute to the redefinition of several diseases from local to systemic pathogenesis by exploring the tissues contribution to common biological fluids via EVs. The scientific community has already been working to develop new methodologies capable to properly address this issue. On one hand, one approach consists of identifying particular EVs surface markers specific of a certain cell-type. For instance, Uil et al. investigated miRNA profiles of circulating EVs in different stages of diabetic nephropathy focusing on the miRNA content of activated platelet- (Cd61-positive) and not activated platelet- (CD62p-positive) derived EVs, not activated endothelial (CD34-positive) cell- and activated endothelial (CD34e-positive) cell-derived EVs, erythrocyte- (CD235a-positive) derived EVs [116]. Ohmichi et al. develop a particular method for the isolation of brain-derived exosomes (BDEs) from blood, such as neuron- (SNAP25 positive), astrocyte- (EAAT1-positive) and oligodendrocyte- (OMG-positive) derived exosomes (CD81-positive), allowing to identify potential biomarkers associated with CNS-diseases [117,118]. Another example is given by Svenningsen et al. who performed a cross-analysis between proteomic data of urinary EVs (uEVs) and tissue RNA expression data in order to explore the potential parental cells both within and outside the urinary tract [119]. Another innovative study performed by Jones et al. investigated the origin of blood microbiota in patients with inflammatory bowel disease, by isolating bacterial EVs (BEVs) from plasma, extracting their BEV-associated DNA and performing 16S rRNA gene sequencing [120]. On the other hand, another approach consists in relying on EVs content profiles to trace EVs back to their origin. Larssen et al. performed an interesting study by the affinity-based proximity extension (PEA) assay with the purpose of characterizing exosomal proteins from different sources and attempting to identify their cellular origin. They applied multiplex PEA for proteomic profiling of seven different cell lines and their released exosomes, as well as of exosomes from breast milk and seminal plasma. The findings show that the protein composition of each cell lysate clusters close to the one of their respective released exosomes, once only common identified proteins were considered, and the protein compositions of exosomes isolated from breast milk and seminal plasma interestingly clustered close to the breast cancer cell line and the prostate cancer cell lines, respectively. As a result, exosomes could be dated back to their progenitor cells by their protein signature. [121]. Another example of this approach relies on EVs transcriptomics, as showed by the findings of Li et al. They developed a particular method, called “EV-origin”, that enables digital quantification of EVs tissue-cellular source contribution from plasma EVs long RNA sequencing (exLR-seq) profiles [122]. These innovative methods challenge the possibility to trace cellular origin of EVs, thereby they might be useful to identify diagnostic and prognostic biomarkers from non-invasive collected samples. Summary data are reported in Table A1.

## 3. New Perspectives

Along the years numerous efforts have been made to map human genes and to analyze human metabolism, regulatory networks and protein–protein interactions especially, in physiological and pathological conditions. Remarkably, these analyses produced impressive lists of disease–associated molecules, each coming from a single layer of high-throughput “omic” analyses. Indeed, nowadays several EVs databases are available and publicly accessible such as EVpedia, [89], Vesiclepedia [87], Exocarta [88] and EV-TRACK [123]. However, every “omic” layer can only provide limited insights into the biological mechanisms of a disease, not suggesting any information on the interaction with DNA, RNA, proteins and metabolites, frequently playing complementary roles to jointly perform a certain biological function. Such complementary effects and synergistic interactions between various omic layers of a complex biological system can only be observed by an integrative study of the multiple molecular layers. Therefore, the integration of omics data of different origin in a system-wide observation could reflect the intricate interplay among biological variations at different levels of regulation (Figure 5). There are two potential approaches to investigate multi-omics data: the first requires prior knowledge of central molecular pathways driving certain physio-pathological events, while the second starts from no existing knowledge, cross-relating multiple datasets to identify molecules whose alterations might be inter-correlated [124,125]. Remarkably, one very ambitious project that attempts to use integrated multi-omics data for health assessment and prediction is known as “integrated Personal Omics Profiling (iPOP)” project and it consists in the longitudinal profiling of an extended cohort over time with a wide set of omics approaches and clinical sets, with the purpose of collecting parameters and data useful in long-term health management [126]. Another large-scale effort is represented by the Trans-Omics for Precision Medicine (TOPMED) initiative of the National Heart, Lung, and Blood Institute (NHLBI), which attempts to generate more comprehensive omics data for a wide range of study cohorts [127]. In fact, one essential requisite for multi-omics integration is, beyond the generation of different omic datasets from the same biological samples, the development of statistical and annotation tools, essential for the interpretation of data [128,129]. Many examples of multi-omics studies are available in literature, especially applied to biomarker discovery, such as Jiang et al. for pancreatic cancer [130], Zheng et al. for non-small cell lung cancer [131] and Buttacavoli et al. for colon cancer [132]. Other studies extended from drug discovery, such as Sha et al. for diabetic nephropathy [133] and Meng et al. for chordoma [134], to the understanding of pathogenesis, as performed by Wang et al. in clear cell renal cell carcinoma [135] and to the establishment of accessible databases of clinical and/or multi-omics data, as Answer ALS [136] or Fibromine [137]. Recently, this multi-omic approach has also been applied to the developing single-cell technology, as it could allow to capture multiple omic layers from the same cell. For instance, Unterman et al. performed a multi-omics single-cell analysis to investigate the immune responses in hospitalized patients with stable or progressive COVID-19 and the cellular effects to tocilizumab [138]. Of interest, single-cell multi-omics might make it easier to establish associations between variations in multiple omic layers within the same single cells, as defined relationships are unambiguous. Furthermore, it also enables to study specific actions of individual cells in a spatially organized context, suggesting that the spatial information might be essential to the understanding of the whole picture [139]. Since the nature of EVs, such integrative multi-omics data analysis could be applied to accurately explore EVs functions. While modern high-throughput technologies and methodologies enable to obtain reliable EV omic data, adequate bioinformatics tools to be used for the integration and the functional interpretation of these datasets in cross-relation are still few and continuously evolving. For example, the two most approachable data integration methods for EVs study might be the correlation-based method and the network-based method. The former can be used to analyze the relationship between two sets of omic datasets, such as genomics and transcriptomics or transcriptomics and proteomics, although it is limited by the bidirectional modulation of biological information, not taking into account correlations between indirectly associated omic data, such as genomics and metabolomics, for instance. The latter, the network-based method, consists of modelling complex biological interactions in which nodes are represented by biomolecules from EVs content (genes, transcripts, proteins lipids and others) and edges represent the interactions between them. These inferred connections are based on prior knowledge, such as “ontologies” [6]. Nonetheless, still many improvements are needed, from sample collection, standardization of sample processing and EVs isolation to reproducibility of omic analysis and pre-processing of omic data. Summary data are reported in Table A1.

## 4. Conclusions

Given the numerous potentialities of EVs in terms of source of biological knowledge and of translational applications, omic studies represent the optimal approaches to know deeper these nanoscale biological systems. Surely, the forward steps will be directed to the development of innovative computational and bioinformatic tools that would provide the means to integrate and biologically interpret EVs heterogeneous content. Still, many issues are encountered in each step of EVs multi-omic analysis, starting from EVs isolation to the data integration methods, suggesting that this field is at its naïve state and requires further improvements.

## Appendix A

**Table A1:** Summary table of omics sciences in EVs research, their main applications, major reached outcomes and limitations.

Omic Science	Applications	Major Outcomes	Limitations	Reference
**Proteomics**	Biomarkers discovery of EVs subpopulations	Differentiation of small and large EVs populations 2DE vesicular proteomic profile as unique signature Overcome of limit detection of protein biomarker in common biological fluids Customization of chemotherapy drugs and novel therapeutical agents’ delivery and action	Limited knowledge of heterogeneous EVs subtypes Lack of standardized methods of EVs isolation and enrichment	[51,52,53,54]
	EVs Proteomic Profiling and EVs origin			[55,56,57,58,59,60,61,62,63,121]
	Diagnosis, prognosis and progression disease biomarkers			[66,67,68,69,70,71,72,73]
	Optimization of EVs as drug delivery system			[75,76]
**Transcriptomics**	Characterization of EVs RNA content	Detection of a wide variety of RNA species in EVs, especially non coding RNAs Correlation of EVs RNA species with disease diagnosis and progression Identification of tissue-specific EVs RNA expression profiles Improvements in tracing EVs cellular origin	Limited knowledge of gene-regulatory activities of EVs RNA cargo	[77,78,79]
	Diagnostic and prognostic biomarkers discovery			[80,81]
	EVs non-coding RNA s as potential biomarkers of disease			[83,84,85,86]
	EVs origin			[117,120,122]
**Metabolomics**	Biomarkers discovery	EVs metabolome reflects progenitor’s cellular state acting as optimal candidate source of biomarkers Findings of EVs metabolical activity in shaping the extracellular microenvironment	Limited metabolomics data included in common EV databases Lack of standardized methods of sample production, preparation and detection	[91,92,93]
	EVs as metabolically active machines			[94,95]
**Lipidomics**	EVs biogenesis	Increasing information about EVs packaging mechanisms Findings of the involvement of lipid metabolism	Limited knowledge of EVs lipid composition and functions	[96,97,98,99]
	Lipid dysregulations in pathogenesis			[100]
	Biomarkers discovery			[93,101]
**Genomics**	Genomic profiling of EVs	Liquid biopsies for the diagnosis and prognosis assessment of diseases as EVs genetic cargo reflects the mutational status of parental cells gene therapy	Methodological limits in EVs enrichment and purity	[102,103,104,105,106]
	EVs engineering			[107,108]
**Surfaceomics**	EVs surfaceome profiling for diagnostic and therapeutic purposes	Potential candidate surface proteins for tissue-specific EVs enrichment Targeted delivery of engineered EVs for therapeutical purposes	Assessment of specificity and sensitivity of candidate EVs surface markers of target delivery	[109,110,111,112,113,114]
	EVs origin			[116,117,118]
**Multi-omics**	Biomarker discovery	Collection of molecular and clinical data at different –omic levels from an extended cohort useful for long-term health management Establishment of accessible databases of clinical and multi-omic data associated to specific diseases Improvements in multi-omic data association by single-cell approach Development of more comprehensive statistical and annotation tools for the interpretation of data	Difficulties in the association of different omic data due to not adequate bioinformatic tools Lack of standardization of EVs collecting and omic processing methods	[130,131,132]
	Therapeutical targets discovery			[133,134]
	Pathogenesis of disease			[135]
	Databases			[126,127,136,137]
	Single-cell technology			[138]
	EVs origin			[119]
